# Simultaneous Estimation of Low- and High-Order Functional Connectivity for Identifying Mild Cognitive Impairment

**DOI:** 10.3389/fninf.2018.00003

**Published:** 2018-02-06

**Authors:** Yueying Zhou, Lishan Qiao, Weikai Li, Limei Zhang, Dinggang Shen

**Affiliations:** ^1^School of Mathematics, Liaocheng University, Liaocheng, China; ^2^College of Information Science and Engineering, Chongqing Jiaotong University, Chongqing, China; ^3^Department of Radiology and BRIC, University of North Carolina at Chapel Hill, Chapel Hill, NC, United States; ^4^Department of Brain and Cognitive Engineering, Korea University, Seoul, South Korea

**Keywords:** functional connectivity, high-order network, matrix variate normal distribution, mild cognitive impairment, disease diagnosis

## Abstract

Functional connectivity (FC) network has been becoming an increasingly useful tool for understanding the cerebral working mechanism and mining sensitive biomarkers for neural/mental disease diagnosis. Currently, Pearson's Correlation (PC) is the simplest and most commonly used scheme in FC estimation. Despite its empirical effectiveness, PC only encodes the low-order (i.e., second-order) statistics by calculating the pairwise correlations between network nodes (brain regions), which fails to capture the high-order information involved in FC (e.g., the correlations among different edges in a network). To address this issue, we propose a novel FC estimation method based on Matrix Variate Normal Distribution (MVND), which can capture both low- and high-order correlations simultaneously with a clear mathematical interpretability. Specifically, we first generate a set of BOLD subseries by the sliding window scheme, and for each subseries we construct a temporal FC network by PC. Then, we employ the constructed FC networks as samples to estimate the final low- and high-order FC networks by maximizing the likelihood of MVND. To illustrate the effectiveness of the proposed method, we conduct experiments to identify subjects with Mild Cognitive Impairment (MCI) from Normal Controls (NCs). Experimental results show that the fusion of low- and high-order FCs can generally help to improve the final classification performance, even though the high-order FC may contain less discriminative information than its low-order counterpart. Importantly, the proposed method for simultaneous estimation of low- and high-order FCs can achieve better classification performance than the two baseline methods, i.e., the original PC method and a recent high-order FC estimation method.

## Introduction

Functional connectivity (FC) network, calculated by resting-state functional magnetic resonance imaging (rs-fMRI) (Liu et al., 2008), has become an increasingly useful tool for understanding the working mechanism of the brain and providing informative biomarkers for diagnosing some neural/mental disorders, such as autism spectrum disorder (Wee et al., 2016b; Li et al., 2017), major depressive disorder (Greicius et al., 2007; He et al., 2016), obsessive compulsive disorder (Admon et al., 2012), schizophrenia (Zhou et al., 2007; Ganella et al., 2016), social anxiety disorder (Liu et al., 2015a,b), Alzheimer's disease (Zhu et al., 2015; Wang et al., 2017), and its early stage, i.e., mild cognitive impairment (MCI) (Wee et al., 2012; Yu et al., 2017).

In view of its great potential, how to construct high-quality FC networks comes to a key issue. Theoretically, we can treat the FC network as a graph, where the nodes correspond to different brain regions or, more generally, the regions-of-interest (ROIs), while the edges correspond to the pairwise FCs between these nodes. In other words, FC network can be seen as a combination of the node set and the edge set. Currently, researchers have proposed a series of FC network modeling methods (Smith et al., 2011, 2013), among which Pearson's Correlation (PC) is the simplest and the most popular way. Although it has been successfully applied in FC estimation, PC can only capture the low-order (or second-order) statistical information by calculating the pairwise correlations between the network nodes (e.g., ROIs in this paper).

In practice, some high-order statistics (e.g., the correlations among different edges) may also offer additional and useful information for FC analysis (Plis et al., 2014; Chen et al., 2016; Zhang et al., 2016). To make this easy to understand, we consider the traffic network as an analogy, where the cities are regarded as nodes, the roads are edges between the city nodes, and the traffic flow can be used as a measure of the dynamic weight on each edge. In this example, the road (or traffic flow) provides the low-order connection information of the network. On the other hand, however, there may exist some relationships among different roads/edges. For instance, a traffic jam on one road tends to affect the traffic flow on another road. Compared to the edges that measuring the relationship between nodes, the relationship between edges is expected to provide some high-order connection information for a network system.

Recently, some high-order methods have been developed for estimating FCs (Plis et al., 2014; Chen et al., 2016, 2017; Zhang et al., 2016). For example, Plis et al. used mutual information to investigate the nonlinear interactions among brain regions (Plis et al., 2014); Zhang et al. proposed a high-order FC network estimation method based on the topographical information and applied it to MCI detection (Zhang et al., 2016); Chen et al. proposed a scheme for estimating high-order FCs based on sliding windows, and empirically verified that it can achieve better accuracy than the low-order counterparts for early MCI identification (Chen et al., 2016). However, modeling such a high-order FC in Chen et al. (2016) involves a consistent amount of parameters, thus easily leading to an over-fitting problem given limited training data. In addition, the work of Chen et al. (2016) is heuristic without support from mathematical models, and thus cannot make it clear where the high-order relationship (i.e., correlation's correlation) is theoretically derived from.

To address these problems, in this paper we put forward a novel high-order FC network estimation method based on Matrix Variate Normal Distribution (MVND) (Gupta and Nagar, 2000). MVND *not only* provides a clear theoretical explanation for high-order FC networks, *but also* achieves both low- and high-order networks simultaneously in a unified framework. In particular, a sliding window approach is first used to generate a sequence of overlapping time subseries. For each subseries, the traditional low-order FC network is constructed by PC. Then, the so-constructed FC networks are used as samples to estimate the final low- and high-order FC networks by maximizing the likelihood function of MVND. Especially, we adopt Kronecker product as a novel way to reduce the number of parameters (see section The Proposed Method for details). To validate the effectiveness of the proposed method, we perform experiments to identify MCI subjects from Normal Controls (NCs), based on the estimated low- and high-order FC networks. The experimental results illustrate that the proposed approach can achieve better performance than the baseline method, and both low- and high-order information are generally helpful for classification. Both preprocessed data and codes can be downloaded in https://github.com/Zhouyy92/high-order-based-on-MVND/. More specifically, the main contributions of our work can be summarized as follows:

1. To our best knowledge, this is the first work that adopts MVND for estimating high-order FC networks. MVND *not only* provides a clear mathematical definition of high-order network, *but also* can simultaneously estimate low- and high-order FC networks in a single model.
2. A new finding of this paper is that the low-order FC tends to contain more discriminative information than its high-order counterpart, which is exactly the opposite conclusion of Chen et al. (2016). However, similar to Chen et al.'s work, we also note that the fusion of low- and high-order FCs can generally improve the identification accuracy to some extent, indicating that there is some useful information in the high-order FCs for discrimination.

The rest of this paper is organized as follows. In section Materials and Methods, we introduce the materials and propose our method. In section Results, we evaluate the proposed method in identifying MCI subjects from NCs. In section Discussion, we discuss our findings based on the experimental results. In section Conclusion, we conclude the whole paper.

## Materials and methods

### Data acquisitions and processing

Totally, 137 participants, including 68 MCI patients and 69 NCs from Alzheimer's Disease Neuroimaging Initiative (ADNI)^1^ dataset (Jack et al., 2008), are used in this study. The observed rs-fMRI images were scanned by 3.0T Philips scanners with the following parameters: TR/TE is 3,000/30 mm, flip angle is 80°, imaging matrix size is 64 × 64 with 48 slices and 140 volumes, and voxel thickness is 3.3 mm.

The acquired rs-fMRI data was processed by SPM8^2^ toolbox based on the well-accepted pipeline. The first three volumes of each subject were removed for signal stabilization. Then, the remaining 137 volumes were corrected for different slice acquisition timing and head motion (Poldrack et al., 2011). To further reduce the influences of nuisance signals, regression of ventricle and white matter signals as well as six head-motion profiles were conducted. Based on the Automated Anatomical Labeling (AAL) template atlas (Tzourio-Mazoyer et al., 2002), the pre-processed Blood Oxygen Level Dependent (BOLD) time series signals were partitioned into 116 ROIs. Prior to FC estimation, the mean rs-fMRI time series of each ROI was band-pass filtered from 0.01 to 0.08 Hz. Finally, the mean time series was put into a data matrix **X** ∈ *R*^137 × 116^, which will be used for FC network estimation.

### Functional connectivity network estimation

#### Baseline method

According to a recent review (Smith et al., 2013), PC is the most widely-used method for estimating FC networks. Therefore, in this paper, we select PC as the baseline and building block for developing our method, even though the idea can *in principle* be used in any correlation-based FC construction methods. The PC-based FC is defined as follows:

(1) $$\begin{array}{l} W_{ij}^{\left(PC\right)}=\frac{{\left(x_{i}-{\overset{-}{x}}_{i}\right)}^{T}\left(x_{j}-{\overset{-}{x}}_{j}\right)}{\sqrt{{\left(x_{i}-{\overset{-}{x}}_{i}\right)}^{T}\left(x_{i}-{\overset{-}{x}}_{i}\right)}\sqrt{{\left(x_{j}-{\overset{-}{x}}_{j}\right)}^{T}\left(x_{j}-{\overset{-}{x}}_{j}\right)}} \end{array}$$

where $x_{i}\in R^{V}\left(i=1,2,\cdots \;,P\right)$ is the time series associated with the *i*th ROI, *V* = 137 is the total number of temporal image volumes, *P* = 116 is the number of ROIs, and ${\overset{-}{x}}_{i}\in R^{V}$ is the corresponding mean vector of *x*_*i*_. Without loss of generality, in this paper we suppose that *x*_*i*_ is centralized by $x_{i}-{\overset{-}{x}}_{i}$ and normalized by $\sqrt{{\left(x_{i}-{\overset{-}{x}}_{i}\right)}^{T}\left(x_{i}-{\overset{-}{x}}_{i}\right)}.$ Then, PC can be simply expressed as $W_{ij}^{\left(PC\right)}={x_{i}}^{T}x_{j}$, or, an equivalent matrix form,

(2) $$\begin{array}{l} {\text{W}}^{PC}={\text{X}}^{T}\text{X} \end{array}$$

where $\text{X}=\left[x_{1},x_{2},\cdots \;,x_{P}\right]\in R^{V\times P}$ is the data matrix.

Given the fact that the BOLD time series signals commonly contain noises, the original PC-based FC network tends to be dense (Fornito et al., 2016). To alleviate this problem, the thresholding operation is generally employed to filter out the noisy or weak connections. Please refer to, for example, section 3.2.1 in Fornito et al. (2016) for a detailed discussion on different thresholding schemes.

#### The proposed method

In this section, we introduce the new FC estimation scheme based on MVND that can encode both low- and the high-order correlations in a single framework. As a result, we can model FC from two different views.

**Figure 1:**
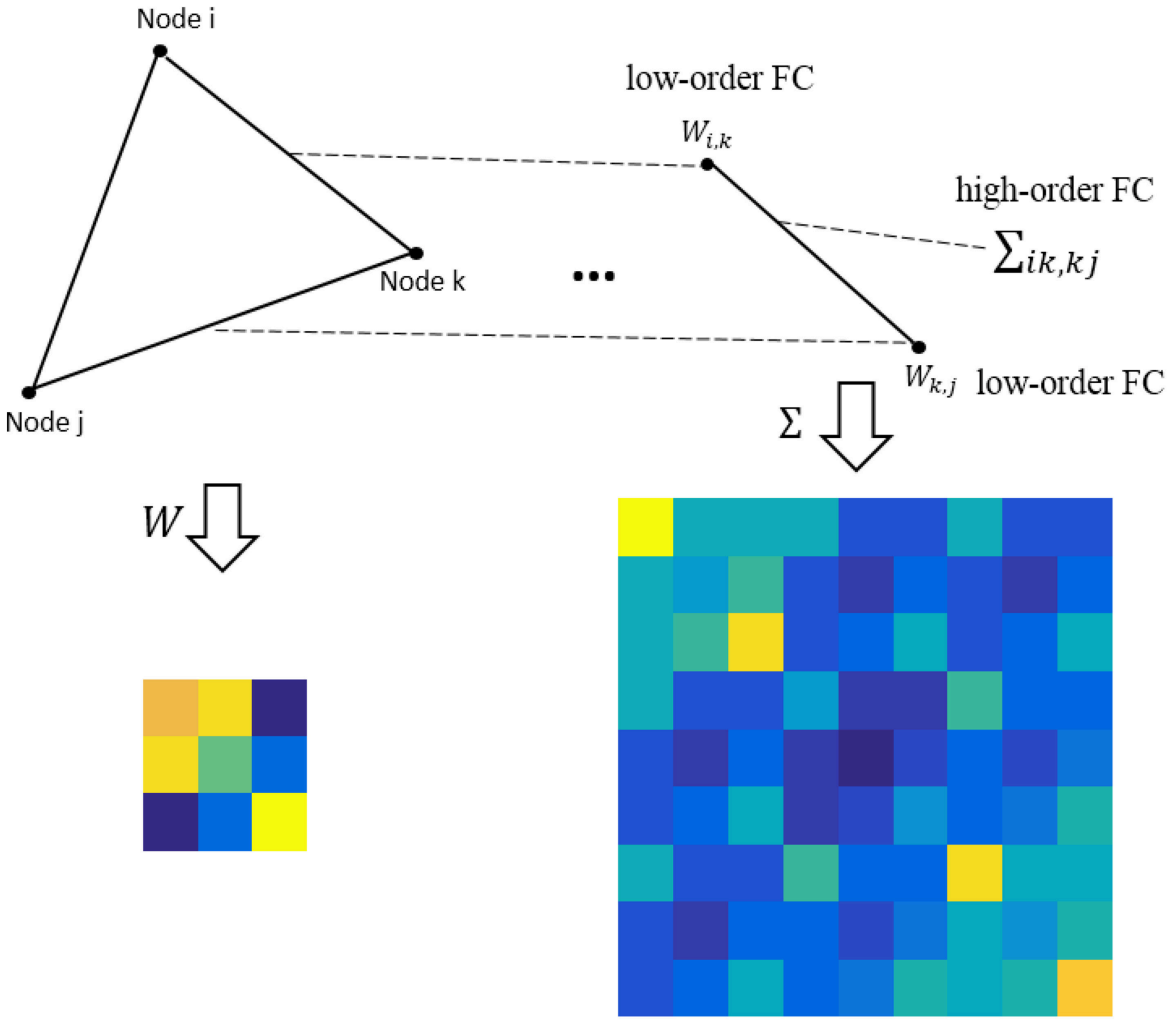
An intuitive explanation for the low- and high-order FC. Note that the low-order FC measures the traditional correlation between nodes, while the high-order FC measures the correlation between edges (i.e., the correlation's correlation).

In particular, we suppose the low-order FC between the *i*th and *j*th ROIs is a random variable *w*_*ij*_ that follows the normal distribution, and thus the corresponding FC network is a random matrix W = (_*w*_*ij*_)*P*×*P*_ that has the multivariate normal distribution. That is,

(3) $$\begin{array}{l} W\sim N\left(\text{M},\Sigma \right) \end{array}$$

where **M** ∈ *R*^*P*×*P*^ is the population mean or mathematical expectation of W, and **Σ** ∈ *R*^*P*^2^^ × *P*^2^ is the population covariance matrix of W. Note that the entries in **M** measure the relationship between the network nodes (i.e., ROIs), still corresponding to the low-order FC; while the entries in **Σ** describe the relationship between the edges, corresponding to the higher-order FC, as shown in Figure 1.

Although the **Σ** in Equation (3) gives a clear definition of the high-order FC, the estimation of a *P*^2^ × *P*^2^ matrix is challenging, since it contains a consistent amount of parameters. For example, *P* = 116 in this study can result in *P*^2^ × (*P*^2^ − 1)/2 ≈ 9 × 10^7^ free parameters. In Chen et al. (2016), they first reduce the number of the edges by clustering them into some groups, and then consider these groups as new “nodes” for constructing the high-order FC network. However, such a scheme has no clear mathematical explanation or principled way to determine the cluster size.

Therefore, in this paper we propose a new strategy to eliminate the difficulty of estimating **Σ** by assuming that it has a form of Kronecker product decomposition (Gupta and Nagar, 2000), i.e., **Σ** = **Ω_1_ ⊗ Ω_2_**. In other words, the random network matrix W follows the MVND:

(4) $$\begin{array}{l} W\sim N\left(\text{M},\Omega_{1}⊗\Omega_{2}\right) \end{array}$$

More specifically, the probability density function of MVND is defined (Gupta and Nagar, 2000) as follows:

(5) $$\begin{array}{l} f\left(W\right)={\left(2\pi \right)}^{\frac{-P^{2}}{2}}det{\left(\Omega_{1}\right)}^{\frac{-P}{2}}det{\left(\Omega_{2}\right)}^{\frac{-P}{2}}\\ \times e^{-tr\left(\frac{1}{2}{\Omega_{1}}^{-1}\left(W-\text{M}\right){\Omega_{2}}^{-1}{\left(W-\text{M}\right)}^{T}\right)} \end{array}$$

where $\Omega_{1},\Omega_{2}\in R^{P\times P}$ are positive semi-definite, indicating the column and row covariance matrices of W, respectively. The *det*(·) and *tr*(·) denote the determinant and trace operators of a matrix, respectively. In section Discussion, we will give a further discussion on the meaning of the Kronecker product decomposition in MVND by comparing with clustering scheme in Chen et al. (2016).

In this study, we mainly focus on the *undirected* FC network, meaning that the edge weight matrix W is symmetric, and therefore the corresponding column covariance matrix is equal to the row covariance matrix, i.e., **Ω**_**1**_ = **Ω**_**2**_. Without loss of generality, we let **Ω** = **Ω**_**1**_ = **Ω**_**2**_ for simplifying the mathematical expression. Since the **Ω** has the size of *P* × *P*, the number of the parameters need to be estimated in **Σ** reduces from *P*^2^ × (*P*^2^ − 1)/2 to *P* × (*P* − 1)/2, and the **Ω** includes all information for tracking back **Σ** under the MVND assumption. In what follows, we develop a two-step scheme for estimating the parametric matrices **M** (corresponding to the low-order FC network) and **Ω** (corresponding to the high-order FC network).

##### Steps 1 sampling: construct FC network series by sliding windows

**Figure 2:**
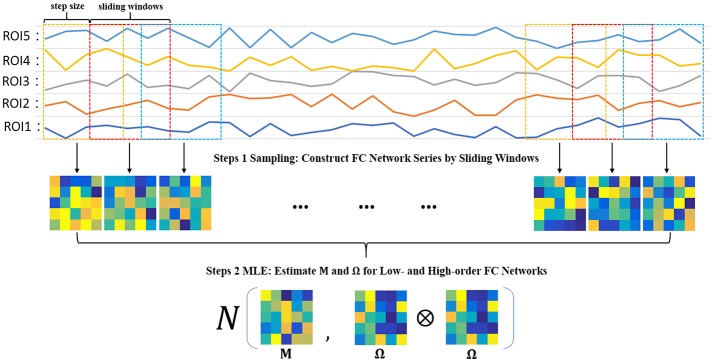
The two-step framework for estimating low- and high-order FC networks.

In order to estimate **M** and **Ω**, we first use the sliding window approach to generate samples, as shown in the top two panels of Figure 2. In particular, we suppose *N* is the width of the sliding windows and *s* is the step size of two adjacent windows, and thus we can obtain *K* = [(*V* − *N*)/*s*] + 1 windows, where *V* is the number of image volumes. Based on the subseries in each window, we calculate the FC networks via PC. More rigorously, we define ${\text{x}}_{i}^{k}\in R^{N},\left(k=1,\cdots \;,K\right)$, to denote the *k*th subseries associated with the *i*th ROI. Then, we concatenate ${\text{x}}_{i}^{k}$ together to obtain a data matrix ${\text{X}}^{\left(k\right)}=\left[{\text{x}}_{1}^{k},{\text{x}}_{2}^{k},\cdots \;,{\text{x}}_{P}^{k}\right]\in R^{N\times P}$ corresponding to the *k*th window. Similar to PC defined in Equation (2) with centralized and normalized procedures, the *k*th temporal FC network **W**^(*k*)^ is constructed as follows:

(6) $$\begin{array}{l} {\text{W}}^{\left(k\right)}={\left({\text{X}}^{\left(k\right)}\right)}^{T}{\text{X}}^{\left(k\right)} \end{array}$$

As a result, we can get *K* samples for estimating **M** and **Ω**.

##### Steps 2 MLE: estimate M and Ω for low- and high-order FC networks

Based on the *K* temporal FC networks as samples, we estimate the low- and high-order FC networks (corresponding to **M** and **Ω**, respectively) by the maximum likelihood estimation (MLE) theory of MVND (Dutilleul, 1999; Zhang and Schneider, 2010). More specifically, the MLE of the mean **M** is

(7) $$\begin{array}{l} M=\frac{1}{K}\sum_{k=1}^{K}W^{\left(k\right)} \end{array}$$

and the MLE of **Ω** can be achieved by the following iteration formula,

(8) $$\begin{array}{l} \Omega =\frac{1}{KP}\sum_{k=1}^{K}(W^{\left(k\right)}-M)\Omega^{-1}{\left(W^{\left(k\right)}-M\right)}^{T} \end{array}$$

Note that, however, the estimation of **Ω** represents the covariance matrix rather than the correlation matrix, and therefore we employ the normalized **Ω** as the high-order FC network in our study. As such, the algorithm for solving Equations (7, 8) is summarized in Table 1.

**Table 1:** Algorithm of MVND-based low- and high-order FC estimation.

Input: *X* //observed data
Output: M and Ω //low- and high-order FC
Apply sliding windows to obtain more samples *X*^(*k*)^ and PC to construct temporal
low-order FC W^(*k*)^ = (X^(*k*)^)*T*X^(*k*)^;
$M=\frac{1}{K}\sum_{k=1}^{K}W^{\left(k\right)}$
Initialize Ω = *I* // identity matrix
while not converge
$\Omega \leftarrow \frac{1}{KP}\sum_{k=1}^{K}\left({\text{W}}^{\left(k\right)}-\text{M}\right)\Omega^{-1}{\left({\text{W}}^{\left(k\right)}-\text{M}\right)}^{T}$
end

### FC network evaluation

In order to verify the performance of the estimated FCs, we utilize the low-order FC network **M** (LoM), high-order FC network **Ω** (HiO), and their fusion (FuMO), respectively, to train classifiers for MCI diagnosis. Of note, FuMO is fused by a linear combination of LoM and HiO. Since it is hard to determine the combination coefficient in practice, in this paper, we simply fuse them by 0.5 × (**M**+**Ω**). In addition, we select the original PC and recent high-order method called HON in Chen et al. (2016), as baseline methods to make a comparison. Based on the estimated FC networks from different methods, we employ the *t*-test (*p* < 0.05) for feature selection, and the linear Support Vector Machine (SVM) (Chang and Lin, 2011) with default *C* = 1 for classification.

Due to the limited subjects, in this paper, we use the nested leave-one-out cross validation (LOOCV) to estimate the classification performance, in which only one participant is left out for testing while the others are adopted for training a classifier and obtaining the optimal parameters. In terms of the thresholding parameter of FC networks, we empirically employ 11 sparsity levels ranging in [1%, 10%, ⋯, 90%, 100%] for all the methods. For instance, 10% means that 90% of the weak edges are filtered out from the FC networks, while 100% means all the edges are reserved. We determine the optimal thresholding parametric value using an inner LOOCV procedure on the training dataset.

## Results

### Classification performance

In our experiments, we adopt accuracy, sensitivity and specificity (Sokolova et al., 2006) as performance metrics to evaluate different FC estimation methods. Their mathematical definitions are given as follows:

(9) $$\begin{array}{l} Accuracy=\frac{TP+TN}{TP+FP+TN+FN} \end{array}$$

(10) $$\begin{array}{l} Sensitivity=\frac{TP}{TP+FN} \end{array}$$

(11) $$\begin{array}{l} \text{S}pecificity=\frac{TN}{TN+FP} \end{array}$$

where *TP, TN, FP, and FN* indicate true positive, true negative, false positive and false negative, respectively.

In Table 2, we first report the best results for MCI identification with specific parametric values, and then, in the next section Sensitivity to Network Modeling Parameters, we discuss the influence of different parametric values on the final classification performance. To be specific, for PC, no free parameter is involved; for the proposed method, the width of the sliding window is *N* = 50, and the step size is *s* = 8; for HON method, *N* = 110, *s* = 1, and the cluster size is 300. As can be seen from Table 2, the proposed method significantly outperforms the two baseline methods.

**Table 2:** Comparison on MCI classification performance with different methods.

**Method**	**Accuracy**	**Sensitivity**	**Specificity**
PC	0.7956	0.7647	0.8261
HON (Chen et al., 2016)	0.8207	0.8194	0.8377
LoM	0.9051	0.9118	0.8986
HiO	0.8394	0.8235	0.8551
FuMO	0.8905	0.8676	0.9130

### Sensitivity to network modeling parameters

**Figure 3:**
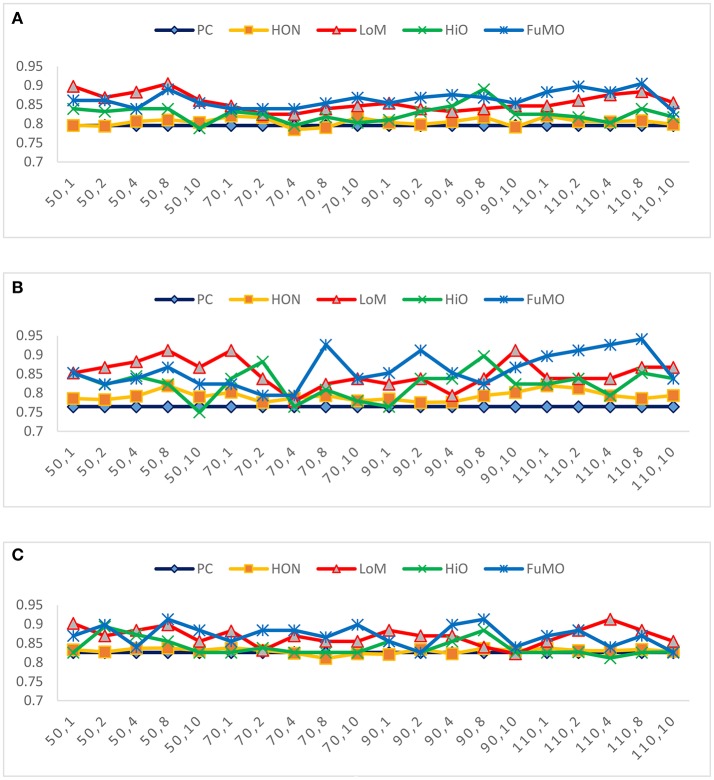
The **(A)** accuracy, **(B)** sensitivity, and **(C)** specificity of each involved method *with respect to* different combinations of window width and step size.

In general, the free parameters involved in the FC network estimation methods have a big influence on the ultimate classification performance. In the proposed framework, there are two free parameters, including the width of sliding windows (*N*) and the step size (*s*). To evaluate the sensitivity of the proposed framework with respect to *N* and *s*, we repeat MCI identification experiments based on different combinations of window width (*N* = 50, 70, 90, 110) and step size (*s* = 1, 2, 4, 8, 10). Figure 3 reports the accuracy, sensitivity and specificity of each involved method *with regard to* different combinations of *N* and *s* shown on the horizontal axis. For example, the “50, 1” denotes that the window width is 50, and the step size is 1.

In Figure 3, we observe that the proposed method consistently outperforms the two baseline methods on most of the parameter combinations. Additionally, compared with conclusion of Chen et al. (2016) that high-order FC networks work better than low-order FC networks, we actually have an opposite finding that low-order FC networks are instead more discriminating than the high-order counterparts under most cases. In the next section, we will further discuss the possible reasons for this new finding.

## Discussion

In this paper, we propose a new FC network estimation framework based on MVND that can simultaneously capture low- and high-order correlation information in data. The proposed method is validated on ADNI dataset by an MCI identification task. According to the experimental results, we have the following discussions:

1. In general, the performance of the proposed method outperforms the baseline methods, including the original PC method and the recently proposed high-order method (HON) in Chen et al. (2016). Compared with PC, the proposed method encodes the temporal information by the sliding window scheme, and integrates the information based on MVND. Since the brain has an inherent dynamic property, an effective modeling of the dynamic temporal information in FC network is expected to improve the final performance (Marusak et al., 2016; Wee et al., 2016a; Liu et al., 2017; Park et al., 2017). In this paper, we simply employ the sliding windows, the most popular method (Hutchison et al., 2013; Preti et al., 2017), to capture dynamic temporal information. However, signals in the sliding windows can be easily influenced by some non-stationary noise, thus resulting in sudden changes of FC across the brain regions (Hutchison et al., 2013); on the other hand, it is a dilemma to choose an appropriate window width or step size, since the window width should be short enough to capture short-term fluctuations while long enough to allow robust FC estimation (Sakoglu et al., 2010). Especially, in terms of short windows, it may contain less cycles of lower frequency, resulting in less stable measures (Wang et al., 2018). Therefore, in our experiments, we investigate different window sizes in a large range, and empirically found that most methods are sensitive to this parameter. In practice, we need to select window width carefully toward better understanding of dynamics in brains.
2. Interestingly, we find that the proposed low-order FC is generally more discriminative than its high-order counterpart, which is contrary to the conclusion in Chen et al. (2016). We argue that our result is relatively reasonable, since the low-order FC (corresponding to the mean of the random variables) determine the main tendency of the data, while the high-order FC (corresponding to the covariance of the random variables) only capture the spread of the data and could be more noisy as well. Of course, the high-order FCs tend to include some informative structures for MCI identification, because, as reported in Table 2 and Figure 3, HiO generally performs better than PC. In addition, the simple combination of low- and high-order FC networks (i.e., FuMO) can achieve better performance than a single-view network. More specifically, as shown in Figure 3, it has 60% possibility for FuMO to obtain the best accuracy, while 55 and 60% possibilities for sensitivity and specificity, respectively.
3. From the experimental results, we also find that the estimated FC networks (including the low-order, high-order, and their combination) consistently outperform HON in Chen et al. (2016). In what follows, we discuss the similarities and differences between HON and our method, aiming to help understand why their method does not work well. In particular, the two methods share the same sliding window step for generating network samples. For the high-order FC network estimation step, however, HON method applied the clustering algorithm to divide the edges of a network into different groups for reducing the computation/estimation cost. Such a scheme actually destroys the original structure of the network, and also introduces a new free parameter (i.e., cluster size) that can cause over-fitting problem. Additionally, HON is heuristic without a support of mathematical theory, and thus cannot provide a clear definition of the high-order correlation. In contrast, based on the MVND assumption, our proposed method actually takes the edges linked to the same node (i.e., ROI) as a cluster, i.e., 116 clusters in this study, which *not only* keep the natural structure of the network, *but also* avoid the issue of selecting cluster size.

## Conclusion

In this paper, we develop a novel PC-based FC estimation framework with the assumption that the network edge weights follow the MVND. The proposed method is simple, has a rigorous mathematical model, and can capture both low- and high-order FCs of the brain network simultaneously. The experiments on MCI identification show that our proposed method outperforms the original PC method and a recent high-order FC estimation method. On the other hand, although we design our method based on PC, the idea can be used in any correlation-based FC estimation methods. In the future, we plan to generalize the MVND-based scheme to the partial correlation-based FC estimation problem.

## Author contributions

DS: Proposed the idea of high-order FC and provided the preprocessed rs-fMRI data; LQ: Proposed the mathematical models for simultaneously estimating low- and high-order FC networks; YZ, LZ, and WL: Designed the procedures of MCI evaluation experiments; All authors developed the estimation algorithm and contributed to the preparation of the article, figures, and charts.

### Conflict of interest statement

The authors declare that the research was conducted in the absence of any commercial or financial relationships that could be construed as a potential conflict of interest.

## Footnotes

^1^http://adni.loni.ucla.edu

^2^http://www.fil.ion.ucl.ac.uk/spm/
